# Early supraclavicular nerve grafting or distal nerve transfer for restoration of elbow flexion in incomplete adult traumatic brachial plexus injuries

**DOI:** 10.1016/j.bas.2026.106113

**Published:** 2026-06-01

**Authors:** Justus L. Groen, Simon Miedema, Yongxi Long, Erik W. van Zwet, Martijn J.A. Malessy, Willem Pondaag

**Affiliations:** a Leiden Nerve Center, Department of Neurosurgery, Leiden University Medical Center, Leiden, the Netherlands; b University Neurosurgical Center Holland, Leiden University Medica Center, Haaglanden Medical Center and HAGA Teaching Hospital, Leiden and the Hague, the Netherlands; c Biomedical Data Sciences, Leiden University Medical Center, Leiden, the Netherlands

**Keywords:** Brachial plexus injury, Upper trunk, Nerve grafting, Nerve transfers

## Abstract

**Introduction:**

Restoration of elbow flexion is the primary treatment goal in adults with supra-clavicular traumatic brachial plexus injuries (ATBPI). To date, many nerve surgeons will initially await spontaneous recovery when dealing with an incomplete brachial plexus injury to allow sufficient recovery in the median and ulnar nerves to enable distal fascicular nerve transfers (DFNT). An alternative strategy is reconstruction by supraclavicular nerve grafting, which should be performed as early as possible after trauma to minimize denervation time.

**Research question:**

Does early supraclavicular grafting yield equivalent elbow flexion power to DFNT in adults with incomplete ATBPI?

**Methods:**

A retrospective single-centre cohort included adults with C5–C6, C5–C7 or C5–C8 traction lesions treated surgically within 12 months of injury (2009–2021). Lesion extent was defined by clinical, radiological, and intra-operative findings. Patients underwent nerve reconstruction aiming at biceps reconstruction with either nerve grafting or transfer; the reconstruction strategy considered patient age, delay, gap length and stump quality. The primary outcome was biceps strength at ≥2-year follow-up or earlier when a biceps MRC grade 4 was reached.

**Results:**

A total of 164 consecutive surgically treated ATBPI patients were identified, of which 84 met inclusion criteria (C5–C6 n = 30, C5–C7 n = 26, C5–C8 n = 28). Nerve grafting was performed in 21 and DFNT in 52 patients; 11 received other reconstructions. All supraclavicularly explored lesions showed root avulsions or Sunderland grade IV–V damage. DFNT produced MRC 4 outcomes, but the proportion achieving at least useful elbow flexion (MRC 3–4) was similar between DFNT and grafting. Lesion extent influenced both treatment choice and outcome, with DFNT used mainly in C5–C6/C5–C7 injuries and grafting favoured in more extensive C5–C8 lesions. In the grafting group, each additional month of delay reduced the odds of a better MRC category by about 50% (OR 0.49, 95%CI 0.29–0.83); for DFNT, timing was not significantly associated with outcome.

**Conclusions:**

Early supraclavicular grafting, typically used in more extensive lesions, yields lower proportion of MRC 4 biceps strength than DFNT, yet comparable rates of useful elbow flexion (≥MRC 3). Nerve grafting should be performed early, as it is more time-sensitive than DFNT, but offers reconstructive advantages in extended upper trunk injuries.

## Highlights

• Early supraclavicular nerve grafting, although used in more extensive C5–C8 traction lesions, achieves similar rates of useful elbow flexion (MRC ≥3) as distal fascicular nerve transfers.
• Distal fascicular nerve transfers more frequently restore strong biceps power.
• Supraclavicular nerve grafting is more time-sensitive than distal fascicular nerve transfers but offers reconstructive advantages in (extended) upper trunk lesions.
• Each month of delay to supraclavicular grafting halves the odds of achieving a better MRC elbow flexion outcome.

## 1 Introduction

Adult traumatic brachial plexus injuries (ATBPI) result from forceful traction widening the neck–shoulder angle, with severity determined by the magnitude and direction of the applied forces (Narakas, 1980). Mild traction injuries may recover spontaneously, but recovery will not occur in severe nerve injuries with spinal nerve rupture or root avulsion. Clinically, ATBPI can be divided into incomplete lesions, with preserved hand function (upper trunk and extended upper trunk, eUT), and complete lesions producing a flail arm. Restoration of elbow flexion is the primary objective of nerve reconstruction, followed by reinnervation of shoulder and wrist stability, digital flexion, and hand sensation (Weekes et al., 2025).

In eUT lesions, many surgeons delay intervention for several months to allow spontaneous recovery (Midha and Grochmal, 2019). If elbow flexion has not returned after up to 6 months, distal fascicular nerve transfers (DFNT) are usually preferred. DFNTs are performed near the target biceps and brachialis muscles, shortening the regeneration distance, and allowing relatively rapid reinnervation. An alternative approach is supraclavicular nerve grafting early after injury, aimed at minimizing denervation time. Early grafting is only appropriate when the severity of the lesion prevents spontaneous recovery. Early supraclavicular grafting may offer advantages over DFNT, as it provides definitive diagnosis and prognosis. Both techniques aim to reinnervate the biceps and brachialis, but grafting potentially reinnervates more muscles, such as the coracobrachialis, brachioradialis and supinator muscle, restore sensation in the C6 dermatome, and potentially reduce neuropathic pain. Furthermore, supraclavicular grafting avoids donor morbidity from harvesting ulnar and median nerve fascicles required for DFNT. Regeneration distance and patient-related factors, especially age and denervation time, are key determinants of outcome beyond graft length. All patients in this study were adults, but the cohort encompassed a broad age range, and age is known to influence peripheral nerve regeneration and susceptibility to muscle atrophy and sarcopenia (Porche et al., 2025). In our practice, candidates for early nerve reconstruction are selected with a stepwise algorithm that considers patient age, stump quality, gap length, and delay from trauma to surgery (Pondaag et al., 2019).

Evidence directly comparing these reconstruction strategies is limited, particularly because outcome data after early supraclavicular grafting is missing. The scientific support to treat patients early with nerve grafting comes mainly from Birch's group (Birch, 2009). Existing reviews suggest a modest advantage of nerve transfers over grafting for elbow flexion, but they combine heterogeneous lesion patterns and include mainly delayed reconstructions (Yang et al., 2012; Texakalidis et al., 2020). It therefore remains unclear whether in a selected group early supraclavicular grafting can achieve elbow flexion outcomes comparable to DFNT. The aim of this study is to evaluate whether early grafting, chosen through predefined criteria, yields recovery of elbow flexion equivalent to DFNT in adults with eUT ATBPI.

## 2 Methods

We performed a retrospective chart review of all patients with either a C5-C6, C5-C7 or C5-C8 lesion type who were surgically treated for SATBPI between 1-1-2009 and 1-1-2021 with a follow up of 2 years or more, or recovery to MRC grade 4 before this time. We only included patients operated in one year after trauma. The ‘lesion extent’ or ‘lesion severity’ was determined by physical examination, combined with MRI myelogram and perioperative findings. In C5-C6 lesions (UT), shoulder function and elbow flexion were absent, but elbow extension and wrist and hand function were intact. The sensation of C7-T1 dermatomes was intact. In extended upper trunk lesion (eUT) involving C5, C6, C7, elbow extension and wrist extension was additionally weak or absent and sensation of C8-T1 intact. In C5, C6, C7, C8 lesions, the finger flexion was weak as well and no sensation present in the hand. In all patients, reanimation of biceps and brachialis muscle function was the primary target of surgery via supraclavicular nerve grafting or DFNT (single or double Oberlin transfer). The results of biceps force were assessed using the Medical Research Council (MRC) motor grading scale. The study protocol was approved by the Medical Ethics Committee Leiden The Hague Delft.

### 2.1 Patient selection

Patients were eligible for early surgical intervention if the following criteria were met: 1) trauma resulted from high-velocity impact injury; 2) neurological deficit was concomitant with external signs such as supraclavicular bruising pointing to supraclavicular trauma; 3) neurological extent of the lesion befitted at least spinal nerves C5 and C6; and 4) deafferentation pain or Horner sign befitting root avulsion. In the case of such lesions, prompt MRI myelography was performed to identify avulsed root filaments 5) patients were A-B-C stable before nerve reconstructive surgery was considered (Porche et al., 2025).

### 2.2 Surgical technique

A supraclavicular exploration was performed in all ATBPI patients if operated within 6 months after trauma and younger than 60 years of age. The decision to opt for grafting or DFNT to reanimation the biceps brachialis muscle was made during surgery and was based on age of the patient, interval between trauma and surgery, gap length and viability of the proximal stump. To evaluate whether a proximal stump was of sufficient quality to use as an outlet for grafts, MRI myelography was used to exclude a preganglionic lesion. Intraoperatively, the viability of the stump was inspected and stimulated under microscopic magnification (Table 1). Autologous sural nerve grafts were used to bridge the gap between proximal and distal stumps.

**Table 1:** Exploration is performed under microscope magnification ∗not useful for confirming intact proximal stumps if surgery performed in 96 h after trauma, no Wallerian degeneration yet ∗∗useful if surgery was done within 96 h after trauma.

1. inspection of proximal stump and its course into the neuroforamen
2. trimming the proximal stump and check the cross-sectional area for intact fascicular structure and absence of hemorrhage or fibrosis
3. confirm continuity of the dorsal scapular nerve to rhomboid muscle contraction with direct stimulation∗
4. confirm continuity of the C5 and C6 branches of the long thoracic nerve to serratus anterior muscle with direct stimulation∗
5. intra-operative frozen-section examination to check myelin presence and fascicular structure of the proximal stump and exclude intraneural hemorrhages, fibrosis, or neuroma formation
6. direct stimulation of the distal stumps to confirm target muscles∗∗

DFNT was performed if delay was longer than 6 months, in patients >60 years of age and/or the gap was wider than 8 cm or proximal stumps were not viable. For DFNT preferably two fascicles (FCU branch from ulnar nerve to biceps and FCR branch from median nerve to brachialis branch) were transferred. In case of extended upper trunk lesion with strong finger flexion, spared FCU but weakened FCR, we only used one ulnar branch to the biceps muscle. If DFNT was performed for elbow flexion, we performed an intraplexal sensory transfer of C4 to C6 to regain some C6 sensory function.

### 2.3 Outcome

We used the Medical Research Council (MRC) grade of biceps force at last visit as primary outcome of reconstruction. The maximum score was per definition limited to MRC grade 4. We are mainly interested in the association between this outcome and the extent of the injury and the delay until surgery. As we aimed to compare the reconstruction options DFNTs versus early supraclavicular grafting, we collapse Oberlin transfer type 1 and type 2 into category “DFNT” and the remaining transfers into category “Other”. Since both groups are relatively small and quite heterogeneous, we limited ourselves to descriptive statistics.

## 3 Results

From our consecutive series of 164 surgically treated ATBPI patients, 75 patients were complete brachial plexus lesions and 5 were operated after one year delay and therefore excluded. For the current study we analyzed the remaining 84 patients with incomplete lesions: 30 patients had a C5-C6 lesion, 26 patients had a C5-C7 lesion and 28 patients a C5-C8 lesion. At supraclavicular surgical exploration, all nerve lesions were severe (either Sunderland class 4 or 5 or avulsed) and needed reconstruction. In 21 patients supraclavicular grafting was performed: C5 to anterior division of superior trunk (AD-ST) (n = 9); C6 to AD-ST (n = 9); C5 and C6 to AD-ST (n = 3). In 52 patient DFNT was chosen for elbow flexion reconstruction, including 8 monofascicular transfers (ulnar nerve to biceps branch; *Oberlin 1 transfer*) and 44 double nerve transfers (ulnar to biceps, median to brachialis branch; *Oberlin 2 transfer*). Remaining 11 reconstructions strategies for biceps function were 3 intraplexal transfer of fascicles of the anterior filaments of C6 to the proximal C5 stump, 6 intercostal nerve transfer to musculocutaneous nerve (MCN) and 2 medial pectoral nerve transfer to MCN. No complications Clavien-Dindo grade II or higher were encountered (Table 2).

**Table 2:** Characteristics of incomplete ATBPI patients treated with DFNT or supraclavicular grafting. A small group of eleven patients were treated with intercostal, pectoral or intraplexal transfers.

	DFNT	Supraclavicular grafting
Number	52 (single 8; double 44)	21
Male: Female	0,9	0,87
Mean age (range in years)	33,9 (13-78)	35,1 (17-59)
Median delay (range in days)	132 (2-354)	92 (2-221)
Lesion Severity (%)		
C5,C6	23 (44)	5 (23)
C5,C6,C7	21 (40)	4 (19)
C5,C6,C7,C8	8 (15)	12 (57)
Avulsions	75%	73%
Supraclavicular exploration	69%	100%
Graft length cm (range)	NA	4,2 (2,2 - 8)
AE (Clavien-Dindo II or more)	0%	0%

**Fig. 1:**
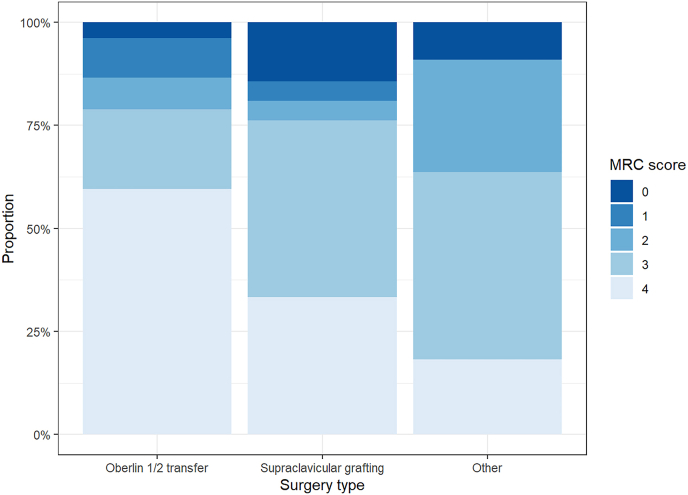
The proportion of elbow flexion MRC outcome by different surgery groups.

After DFNT 31/52 (60%) of patients recovered to MRC 4 and 41/52 (79%) to MRC 3 or 4, compared with 7/21 (33%) MRC 4 and 16/21 (76%) MRC 3 or 4 after nerve grafting. This was statistically significant for MRC 4 (p = 0,042 Chi-square test) but not for MRC 3/4 (p = 0,804, Chi square test).(Fig. 1).

### 3.1 Effect of lesion extent on outcome

**Fig. 2:**
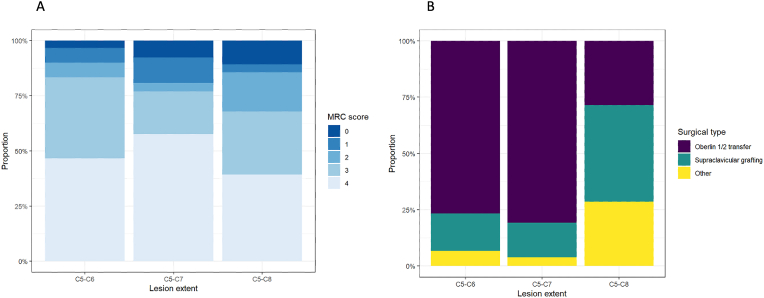
AThe proportion of MRC categories in the three lesion extent groups C5-C6, C5-C7 and C5-C8. MRC0-2 outcome in C5C6 16%, C5-C7 25% and in the C5-C8 32%; differences are not significant: Fig. 2Bthe proportion of surgery types by three lesion extents: DFNT more often in less severe lesions.

Lesion extent reflects the severity of the nerve damage and determines the options for reconstruction. We analyzed the distribution of outcome by lesion extent and the association between lesion extent and surgery type. There was a higher proportion of worse outcomes (MRC 0-2 vs. 3-4) as the lesion extent increased (Fig. 2A). In cases of smaller lesion extent (C5-C6 or C5-C7) DFNT was more often performed. For large lesion extent (C5-C8), supraclavicular grafting was more often performed (see Fig. 2B).

### 3.2 Effect of timing on outcome

**Fig. 3:**
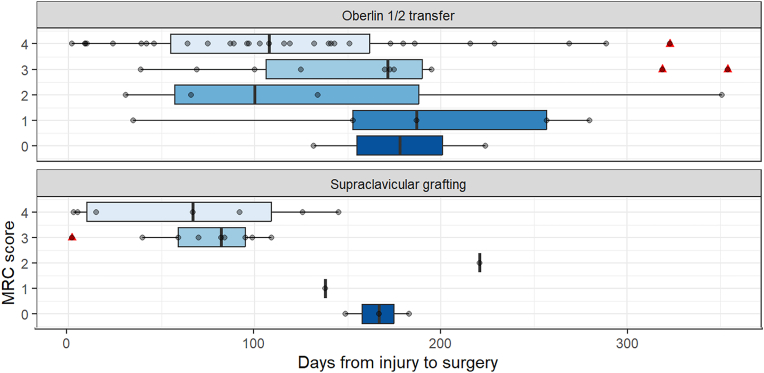
The distribution of the delay time by MRC category, separately for surgery group.

In general, there was a wider spread in the timing of surgery in the DFNT than in the supraclavicular grafting group (Fig. 3). For DFNT, there was no obvious trend that longer delay time was associated with worse outcome. Some patients were operated after delay of 200 days since injury but still had a good outcome (Fig. 2). For supraclavicular grafting, patients with longer delays seemed to have worse MRC outcomes, but this is supported with sparse data, as there are only 5 patients who had MRC 0/1/2 after supraclavicular grafting. We quantified the effect of timing on the MRC outcome using a proportional odds model. This analysis was done separately for the DFNT group and the supraclavicular grafting group. We adjusted for lesion extent, the important prognostic factor we found in our exploratory analysis. There was a significant negative association between delay time and outcome within the supraclavicular grafting group. For each additional month of delay before supraclavicular grafting, the odds of achieving a better MRC recovery category were reduced by 50% (common odds ratio 0.49, 95%CI: 0.29-0.83). There was no significant association between delay time and outcome within the DFNT group (OR 0.85 (95%CI: 0,71-1,01). However, these findings were based on little data, as indicated by the wide confidence interval.

### 3.3 Feasibility of DFNT in C5-C8 lesions

The 28 patients with a C5-C8 lesion were treated with nerve grafting (n = 12), with a DFNT (single fascicular n = 2, double fascicular n = 6) and other transfers (n = 8). At follow up, 5 of the 28 patients with a clinical C5-C8 lesion at presentation did not recover to strong (MRC4 or more) wrist flexion and finger flexion at 6 months after trauma. In these patients a DFNT using flexor carpi ulnaris or flexor carpi radialis nerve fascicles would not have been a viable option. In these 5 patients early grafting was performed for biceps reinnervation, resulting in functional elbow flexion for all patients (MRC3 n = 3, MRC4 n = 2).

### 3.4 Reconstruction of shoulder and hand sensation

Looking at shoulder function, 14 out of 21 patients (67%) with a C5 grafting had reinnervation of the deltoid muscle with useful functional outcome. This useful shoulder outcome was defined as MRC>3 over a range of 60 grades or more of shoulder abduction of anteflexion. Only 3/23 experienced improvement of sensation in C6 dermatome after C6 grafting, with a median follow up time of 99 weeks (IQR 53 weeks), which might be insufficient for the sensory axons to reach the hand.

## 4 Discussion

In this retrospective observational study in incomplete ABPI, we compare the elbow flexion outcome after different surgical techniques in a cohort from a center where early nerve reconstruction surgery is the preferred strategy. In this cohort, DFNT yielded a higher proportion of MRC 4 biceps strength than supraclavicular grafting, whereas the combined rate of “useful” recovery (MRC 3–4) was similar between groups. This pattern aligns with previous systematic reviews in which intraplexal DFNTs, particularly double fascicular Oberlin procedures, more often reach MRC 4 or greater than grafting in upper trunk lesions (Yang et al., 2012; Texakalidis et al., 2020; Garg et al., 2011; Ayhan et al., 2020). In current studies, lesion extent emerged as a major determinant of outcome, with progressively worse MRC grades as the injury extended from C5–C6 to C5–C8, which is consistent with broader literature showing poorer recovery in pan- or near-pan plexus injuries. Importantly, DFNT was used more frequently in the less severe C5–C6/C5–C7 lesions, whereas supraclavicular grafting was preferentially chosen in C5–C8 patterns, introducing inherent selection bias that likely depressed the apparent performance of grafting relative to DFNT. The apparent advantage of DFNT therefore narrows when lesion severity is considered.

Reviews often pool heterogeneous patterns and various reconstructions and timing. Because of this selection pattern, crude comparisons favor DFNT, but stratification by lesion extent in the present study demonstrated that early supraclavicular grafting can provide comparable functional elbow flexion in more challenging C5–C8 cases where DFNT is technically impossible. Often, after C5-C7 and C5-C8 lesions the triceps muscle is not strong enough for DFNT to reinnervate the deltoid muscle (Maldonado et al., 2022). This observation corroborates expert recommendations that in the more extensive ATBPI injuries, supraclavicular grafting must be considered. A benefit of brachial plexus nerve grafting is the increase in number of axons in the lesioned plexus parts, and not to further disperse the intact axons of an already hampered arm. Supraclavicular grafting allowed simultaneous reconstruction of the axillary and radial nerve territories and possibly some recovery of C6 sensation, which aligns with the broader concept that grafting from proximal stumps can reanimate multiple targets beyond elbow flexion, unlike focal DFNT.

The proportional odds analysis showed a significant negative association between delay and MRC outcome within the grafting group, with an approximate 50% reduction in the odds of a better MRC outcome for each additional month of delay. This mirrors extensive evidence that longer denervation periods adversely affect the success of nerve reconstruction, particularly when grafts are used over longer distances. In contrast, no clear timing effect was detected for DFNT in this dataset with a maximum delay of one year, which is in line with large contemporary series suggesting that nerve transfers may tolerate modest delay better than grafting, although even for transfers functional recovery declines when surgery is markedly postponed (Wong et al., 2023). In cases with evidence of severe post-ganglionic injury or root avulsion, reconstruction does not need to be delayed to 6 months; instead, early exploration and reconstruction is advised. Early surgery decreases the reinnervation time of the end organ and early selection of surgical candidates is meaningful and straight forward. In the present series, by using our proposed selection algorithm (Pondaag et al., 2019), we found clear ruptures and root avulsions which required nerve repair in all 84 patients. Therefore, waiting for spontaneous recovery would have been pointless and even hampered them in their chances for meaningful recovery. Prerequisites for early treatment is that the patient is ABC-stable, fit for MRI scanning and timely referred to a specialized nerve center. As ATBPI is rare (Midha, 1997), the latter requires a continues effort of education for referring specialists and staff of the Emergency Departments.

Together with timing and lesion extend, the findings during supraclavicular brachial plexus exploration determine patient individual surgical strategy. These findings support a stratified approach in incomplete ATBPI: DFNT is the preferred option for a patient with an upper trunk lesion with reliable donor fascicles, after longer delay (over 3 months) and at older age (60y or older). Supraclavicular exploration with grafting should be considered in patients with short delay in referral, in C5-C7 and especially in C5–C8 lesion patterns, when viability of donor fascicles for DFNT is doubtful, as it offers simultaneous shoulder reconstruction. Future prospective, ideally multicenter, studies using standardized timing thresholds, lesion grading, and patient-reported outcomes are needed to disentangle the effects of selection bias and to define the precise time window within which early grafting offers equivalent or superior functional benefit compared with DFNT.

## 5 Limitations

This study has several limitations. First, this is a retrospective observational study which leaves room for unobserved confounding and external validation of conclusions are limited by its design. Second, it was subject to selection bias, as we only included patients referred to our national referral center for brachial plexus lesions. Most importantly, our main outcome measure was limited to MRC grade of biceps; endurance, objective outcome measures, as well as function, hand sensation or pain was not systematically assessed.

## 6 Conclusion

Early supraclavicular grafting was used in more extensive lesions and then yields slightly lower maximal biceps strength than DFNT but provides a similar proportion of patients with at least useful elbow flexion (MRC 3–4) across lesion extents. Grafting is more time-sensitive than distal transfers but offers specific advantages in extensive lesions and reconstruction of additional functions. Differences in lesion severity and timing, however, strongly influence outcomes and must be considered when interpreting the findings.

## Declaration of competing interest

The authors declare that they have no known competing financial interests or personal relationships that could have appeared to influence the work reported in this paper.
